# Effect of phosphate and ammonium concentrations, total suspended solids and alkalinity on lignin-induced struvite precipitation

**DOI:** 10.1038/s41598-022-06930-0

**Published:** 2022-02-21

**Authors:** Mozhu Li, Huixin Zhang, Huijuan Sun, Abdul Mohammed, Yang Liu, Qingye Lu

**Affiliations:** 1 Department of Chemical and Petroleum Engineering, University of Calgary, Calgary, AB T2N 1N4 Canada; 2 Department of Civil and Environmental Engineering, University of Alberta, Edmonton, AB T6G 1H9 Canada

**Keywords:** Environmental sciences, Natural hazards, Materials science

## Abstract

To solve the problems of eutrophication and resource crisis, the recovery of phosphorus by struvite (NH_4_MgPO_4_·6H_2_O) precipitation has become a focus of recent research. The feasibility of using Kraft lignin powder as a seed to promote struvite precipitation has been demonstrated in the previous study. In this study, the effect of lignin in promoting struvite precipitation in synthetic wastewater with different characteristics was investigated. Lignin-induced struvite crystallization was tested under various initial concentrations of PO_4_–P and NH_4_–N, total suspended solids (TSS) and alkalinity. At pH 7.9, the enhancement of PO_4_–P recovery remains around 45% under different PO_4_–P and NH_4_–N concentrations. Moreover, lignin is more effective under relatively lower alkalinity and still workable to reduce co-precipitates potential under higher alkalinity. Also, the effect of TSS on PO_4_–P recovery is not significant. Overall, the effect of lignin in promoting phosphorus recovery is relatively stable and can be used in synthetic wastewater with different characteristics.

## Introduction

Phosphorus is a critical raw material for the fertilizer, detergent and pesticide industries, and the demand for phosphorus is met by continuous and intensive mining of phosphate rocks^1–3^. However, phosphate rocks are a limited and non-renewable resource^4^, and their reservation is being depleted^5,6^. A great amount of effort has been made in searching for more sustainable sources to substitute phosphate rocks^7,8^. Meanwhile, significant levels of phosphates are released into wastewater contributing to eutrophication^9–11^. Phosphate-containing wastewater can be a viable resource for phosphorus recovery and recycling^12–14^. Recovering and recycling phosphorus from wastewater is a sustainable approach for phosphorus resource management, and this approach can achieve two benefits simultaneously: prevention of phosphorus enrichment in water bodies and conservation of a finite phosphorus resource^15,16^. Various technologies have been developed to recover and recycle phosphorus from wastewater. These include chemical precipitation, ion exchange, biological removal and recovery, and struvite crystallization^17^. In chemical precipitation, a divalent or trivalent metal salt like aluminum or iron sulphate is added to wastewater to precipitate phosphorus in the form of metal salt sludge, then phosphorus is recovered from the precipitated sludge. The drawback of this technology is that it is very difficult to separate chemically-bonded phosphorus, making phosphorus recovery inefficient^18^. In ion exchange, phosphorus is selectively removed and recovered by using ion exchange media. The application of this technology has been hampered due to: (a) high chemical requirement for the phosphorus recovery, and (b) the performance of the ion exchange media is highly dependent on pH^19^. In biological phosphorus removal and recovery systems, phosphorus in wastewater is assimilated by plants or algae. After harvesting, the plants or algae are processed through specific technologies to release phosphorus. The main disadvantages of this technology are: need for skilled man power and fluctuating performance leading to difficulty in operation^20^. In struvite crystallization, both nutrients (nitrogen and phosphorus) are recovered simultaneously as struvite crystal. Among these technologies, phosphorus crystallization as struvite is the most attractive one^21,22^, as it not only achieves high phosphorus removal from wastewater but also recovers phosphorus in the form of struvite crystal using a simple process. Struvite has a low risk of contamination by pathogens and is a product that can be easily transported and directly applied to soil^23^. Furthermore, struvite is a valuable and excellent fertilizer, because it minimizes the nutrients loss because of its slow releasing rate and its low water solubility^24^.

As for phosphorus recovery and recycling from wastewater as struvite, two approaches have been taken to enhance the efficiency. The first one is optimizing the struvite formation conditions including pH, molar ratio of Mg/P, competing ions and their concentrations, mixing speed, reactor shape and size, and temperature^25^. The second one is adding seed to promote struvite nucleation and crystallization^26^. Our previous study^27^ demonstrates that phosphorus recovery from the waste stream through struvite precipitation using Kraft lignin as the seed is a sustainable approach and a cleaner production process. It has been shown that at relatively low pH of 7.9, using the Kraft lignin as the seed can enhance phosphorus recovery through struvite precipitation by 44.6%, also, lignin addition at pH 7.9 has the advantage of producing high quality struvite crystals by mitigating co-precipitation. This underlines the benefit of using the Kraft lignin as the seed, because producing high purity struvite from wastewater is essential as it can increase the market value of struvite without costly subsequent purification process^15^. Compared with the previously reported seed materials including sand^28^, stainless steel mesh^29^, pumice stone^30^, and biochars^31^, using Kraft lignin as the seed has the following advantages: lignin materials are generated as waste from pulp and paper industries, are biodegradable and environmentally friendly, and are an ideal soil amendment for enhancing plant growth^32^. In addition, Kraft lignin is more efficient in promoting struvite formation^27^.

Phosphorus recovery through struvite formation is a process of chemical crystallization and precipitation^33^, therefore wastewater characteristics, such as pH, ammonium concentration, phosphate concentration, the presence of suspended solids and their concentration, and alkalinity, are possible factors influencing struvite properties^34^. In general, suspended solids and alkalinity were reported to negatively affect struvite formation. For example, Barnes et al.^35^ demonstrated that total suspended solids (TSS) above 1000 mg/L interfered with struvite precipitation process; Desmidt et al.^36^ studied the effect of volatile suspended solids (VSS) on urease driven struvite precipitation and found that struvite crystals could only agglomerate at low VSS concentration; Ping et al.^37^ found that as the concentration of TSS increased, the PO_4_–P recovery efficiency decreased linearly, and the purity of struvite decreased with increasing TSS concentration; Liu et al.^38^ have shown that alkalinity inhibited phosphorus recovery, but the adverse effect of alkalinity on phosphorus recovery had a threshold which was dependent on the initial concentration of PO_4_–P, in addition, alkalinity negatively affected struvite purity; Wei et al.^39^ reported that alkalinity inhibited struvite formation. Recently, it was reported that the increase in TSS led to not only decreased struvite recovery but also lower struvite purity^40^.

Our previous work^27^ has shown the effect of lignin as the seed material which can enhance phosphorus recovery and improve the struvite quality from the synthetical biosolid digestion supernatant. The findings that TSS and alkalinity generally had a negative effect on phosphorus recovery from struvite formation were all reported in wastewater in the absence of any seed material. The objective of this study was to investigate how wastewater characteristics influence struvite precipitation in the presence of lignin as the seed. The wastewater characteristics including PO_4_–P and NH_4_–N concentrations, TSS and alkalinity will be evaluated regarding their impact on the performance of lignin-induced struvite precipitation in terms of phosphorus recovery efficiency and purity of formed struvite. Our study further confirms the effect of lignin in promoting phosphorus recovery from synthetic wastewaters with different characteristics, which widens the scope of using lignin as the seed for promoting phosphorus recovery and recycling from various wastewaters.

## Materials and methods

### Materials

The synthetic wastewaters with different phosphate and ammonium concentrations were prepared using sodium dihydrogen phosphate (NaH_2_PO_4_) and ammonium chloride (NH_4_C1). The alkalinity was adjusted by adding sodium bicarbonate (NaHCO_3_). Magnesium chloride hexahydrate (MgCl_2_·6H_2_O) was added as the magnesium source for struvite. All the reagents were of analytical grade and were purchased from Sigma Aldrich. In this study, the suspended solids were separated from the raw biosolid digestate supernatant by centrifuging at 5000 rpm for 10 min, and then were added to the synthetic wastewater to mimic the different TSS concentrations. The Kraft lignin used in this study was obtained from West Fraser Hinton Pulp (Alberta, Canada), which contains low ash and low sulfur content (0 to 3 wt.%), with a specific gravity of 1.02–1.60. The properties of this Kraft lignin including its particle size distribution at different pH and dosage, X-ray powder diffraction analysis, elemental composition and zeta potential at different pH have been reported in our previous work^27^.

### Struvite precipitation experiments

The struvite precipitation from synthetic wastewaters or the real wastewater was conducted in a beaker with a magnetic stir bar at 200 rpm for 1 min at room temperature, then decreased to 60 rpm for 45 min, and settled for 1 h to precipitate. The supernatant was filtered through a 0.45 μm glass fiber filter to measure the residual PO_4_–P concentration. After centrifuging the suspension samples at 5000 rpm for 10 min, the precipitate was collected by decanting the supernatant, then the precipitate was washed with distilled water by repeated dispersing in water, centrifugation and decanting the supernatant for three times, and finally dried at 40 °C overnight for characterizations. Each experiment was conducted at least in triplicate.

Based on our lignin-induced struvite precipitation study^27^, the optimal conditions are at pH of 7.9 with the Mg/P molar ratio of 1.5 and the lignin dosage of 6 g/L. These conditions enabled the formation of maximum amount and highest purity of struvite. Therefore, 1 M MgCl_2_ was added to adjust the Mg/P molar ratio to 1.5 and 1 M NaOH was used to adjust the pH to 7.9 for this study.

### Chemical analysis and calculation

The PO_4_–P concentration in the solution was measured by Hach methods (TNT844, Hach, USA) before and after the reaction. To measure TSS, water samples were filtered through a 0.45 μm pre-weighted filter. The filter was dried in an oven at 105 °C until the weight of the filter no longer changed and the increase in filter weight representing the mass of the TSS was used to calculate the TSS concentration. The precipitate was analyzed via X-ray diffraction (XRD; Rigaku Ultimate IV, Japan) and scanning electron microscopy (SEM; Zeiss Sigma 300 VP-FESEM, USA) configured with energy dispersive X-ray spectroscopy (EDS; Bruker EDS System, USA). The peaks of the XRD spectra were compared to the Inorganic Crystal Structure Database (ICSD) for struvite confirmation using the reference card PDF #97-006-0626.

### Mineral precipitation modelling

Supersaturation index (*SI*) of various minerals that might be formed together with struvite was calculated by the equilibrium speciation model Visual MINTEQ (ver. 3.0) to predict the potential co-precipitates. The different ion concentrations in feed synthetic wastewater were utilized as model input. *SI* was calculated using Eq. (1):

1 $$ SI = \log \left( {IAP/K_{sp} } \right) $$

where *IAP* is the ion activity product and *K*_sp_ represents the solubility product of the precipitation phase. The minerals only precipitate when *SI* > 0^41^.

## Results and discussion

The influencing factors, including concentrations of PO_4_–P, NH_4_–N, TSS and alkalinity were studied to examine their impact on the performance of lignin-induced struvite precipitation from synthetic wastewater.

### Struvite precipitation from synthetic wastewaters

#### Impact of PO_4_–P concentration on struvite crystallization

**Figure 1:**
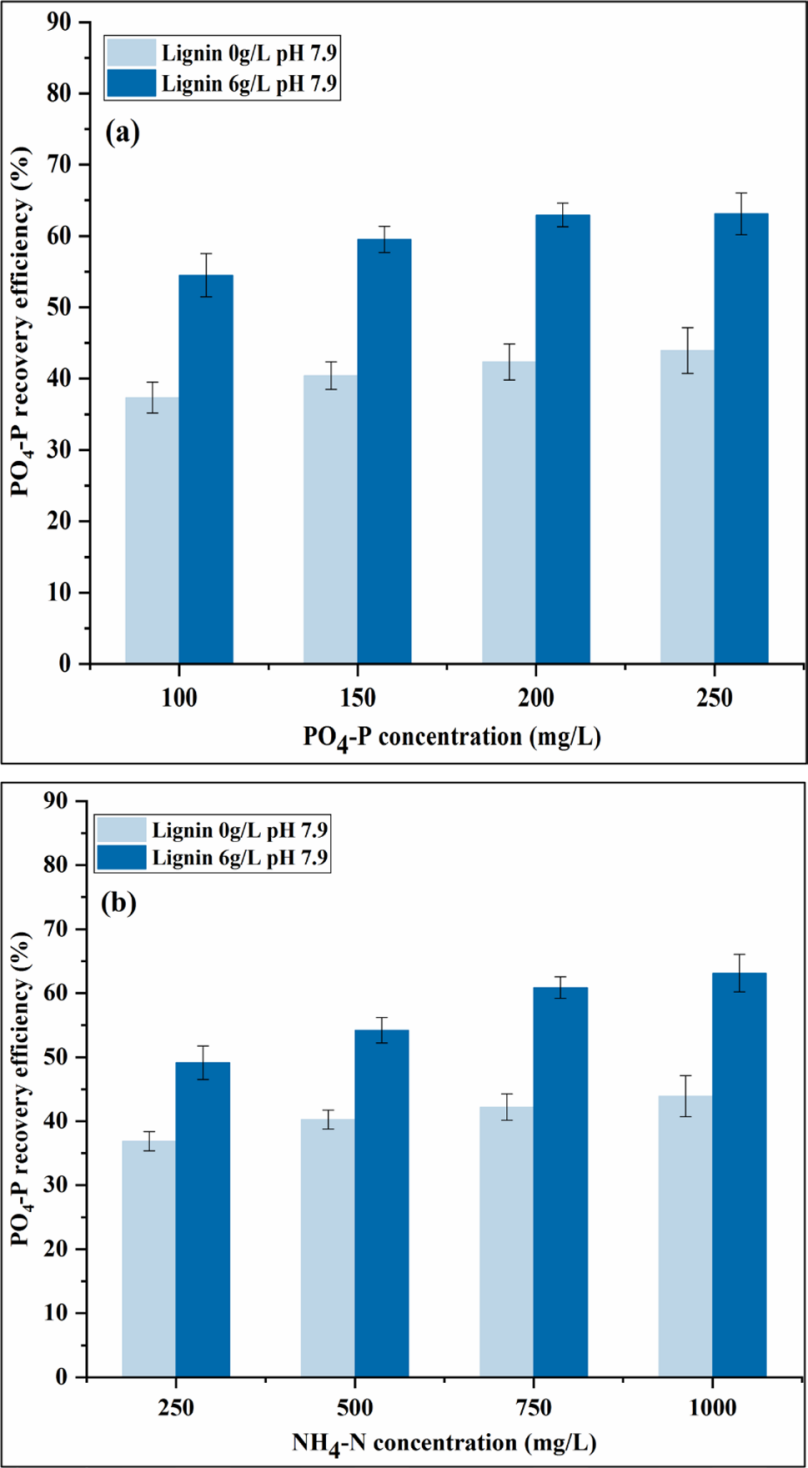
Impact of initial (**a**) PO_4_–P concentration (Mg/P molar ratio = 1.5, [NH_4_–N] = 1000 mg/L) and (**b**) NH_4_–N concentration (Mg/P molar ratio = 1.5, [PO_4_–P] = 250 mg/L) on PO_4_–P recovery efficiency at pH 7.9.

The PO_4_–P recovery efficiencies at different initial PO_4_–P concentrations were examined at the lignin dosage of 0 and 6 g/L. As shown in Fig. 1a, when the initial PO_4_–P concentration is 100 mg/L, the PO_4_–P recovery efficiency is 37.33% at the lignin dosage of 0 g/L, and it reaches 54.51% at the lignin dosage of 6 g/L (two-tailed *t*-test, *p* < 0.05, statistically significantly different). At a higher initial PO_4_–P concentration of 250 mg/L, the PO_4_–P recovery efficiency increases to 43.93% without the lignin addition and 63.13% at the lignin dosage of 6 g/L (two-tailed *t*-test, *p* < 0.05, statistically significantly different), respectively. The PO_4_–P recovery increment as a result of lignin addition from 0 to 6 g/L is all around 45% within the PO_4_–P concentration range studied. Therefore, the use of lignin is effective to enhance phosphorus recovery at different PO_4_–P concentrations.

The influence of initial PO_4_–P concentration on PO_4_–P recovery efficiency is consistent with the previous result that the decrease in PO_4_–P concentration could decrease the phosphorus removal^37^. The struvite crystallization is mainly attributed to the supersaturation index (*SI*) degree of the initial conditions determined by the concentration of all the three struvite constituent ions (Mg^2+^, PO_4_^3−^, NH_4_^+^)^42^. As the concentration of PO_4_–P increases, the degree of supersaturation increases as well, leading to a faster struvite growth rate and consequently, a higher PO_4_–P recovery efficiency.

#### Impact of NH_4_–N concentration on struvite crystallization

As Fig. 1b shows, the PO_4_–P recovery efficiencies at different initial NH_4_–N concentrations were also tested at the lignin dosage of 0 and 6 g/L. The result is similar to that obtained under different initial PO_4_–P concentrations. As the NH_4_–N concentration increases from 250 to 1000 mg/L, the PO_4_–P recovery efficiency increases gradually under both lignin dosages of 0 and 6 g/L (two-tailed *t*-test, *p* < 0.05, statistically significantly different). The addition of lignin is still effective at various NH_4_–N concentrations with the maximal increment of PO_4_–P recovery of 44.6%.

It has been found that higher NH_4_–N concentration not only promotes the PO_4_–P recovery but also increases the purity of the precipitate^43,44^. It is because that the NH_4_–N concentration could influence the supersaturation of the solution and affect the struvite crystallization. Moreover, except for the effect on increasing ionic strength, another possible reason is that excess NH_4_–N is capable of maintaining the pH of the solution because of its buffering capacity, which is conducive to the struvite formation^44^.

Therefore, at a relatively lower pH of 7.9, the effect of lignin on enhancing PO_4_–P recovery efficiency is still significant at different PO_4_–P and NH_4_–N concentrations, which indicates the effect-stability and availability of lignin in promoting struvite crystallization.

#### Impact of alkalinity on struvite crystallization

Afterwards, we prepared the synthetic biosolid digestate supernatant with the different alkalinity to investigate the effect of alkalinity on PO_4_–P recovery.

##### Phosphorus recovery efficiency

**Figure 2:**
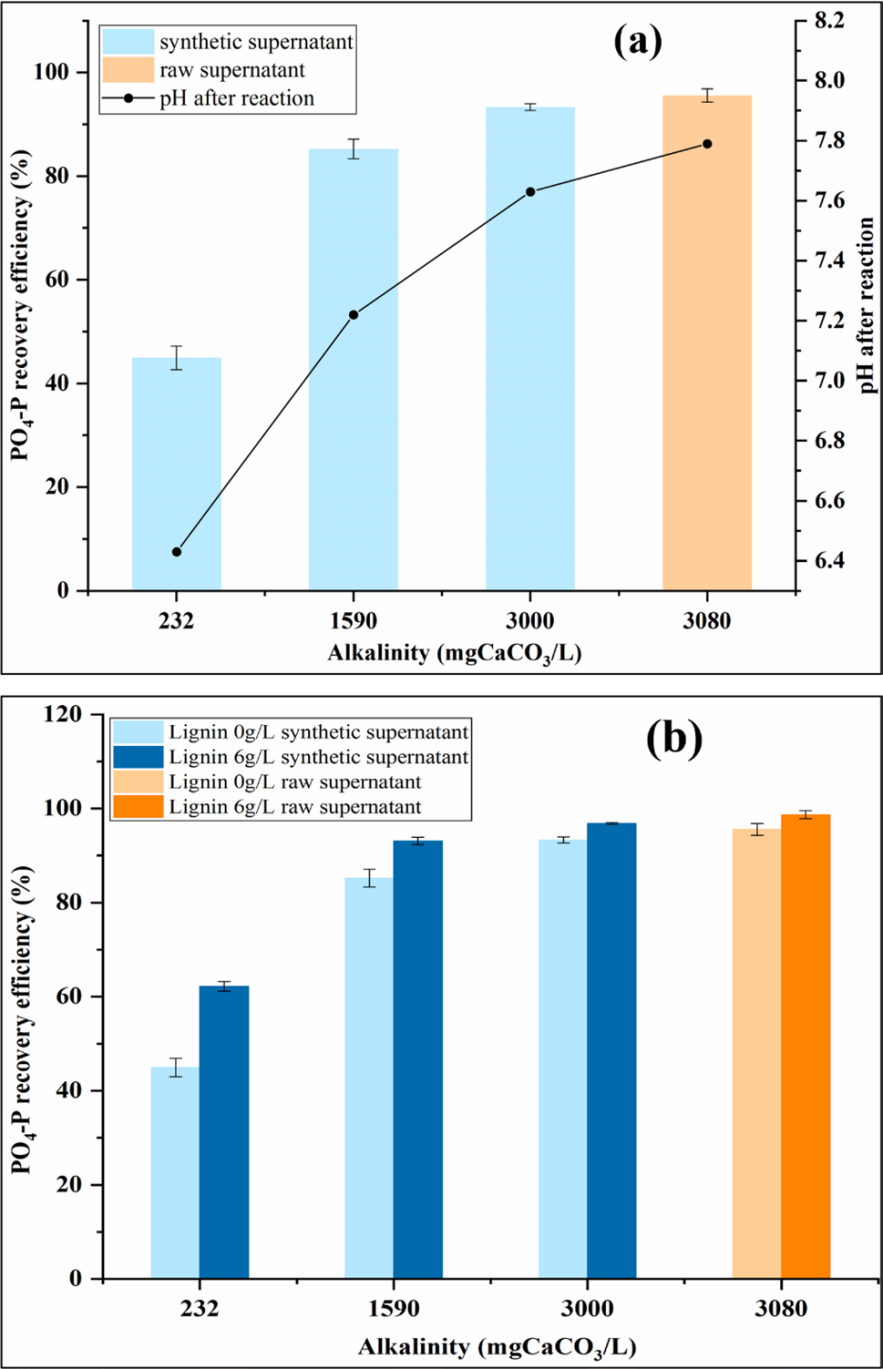
Impact of alkalinity on PO_4_–P recovery efficiency for (**a**) without lignin addition, pH was measured after reaction, (**b**) with lignin addition (synthetic supernatant: initial pH = 7.9, Mg/P molar ratio = 1.5, [PO_4_–P] = 250 mg/L, [NH_4_–N] = 1000 mg/L).

According to Wei et al.^39^, alkalinity is another important parameter affecting PO_4_–P recovery efficiency. Without lignin addition, as Fig. 2a shows, the PO_4_–P recovery enhances significantly as the alkalinity of synthetic wastewater increases gradually. When the alkalinity increases from 232 to 1590 mg/L as CaCO_3_ at the initial pH of 7.9, the PO_4_–P recovery efficiency increases by 40.28% (*p* < 0.05, statistically significantly different). The PO_4_–P recovery efficiency of the synthetic supernatant under 3000 mg/L alkalinity as CaCO_3_ is higher than 95%, which is similar to the PO_4_–P recovery efficiency of the real supernatant (with 3080 mg/L alkalinity), so alkalinity may be the main reason for the high PO_4_–P recovery efficiency of the real lagoon supernatant. The pH of the supernatant after the reaction was measured and the results are shown in Fig. 2a. The pH after the reaction is 6.43 and 7.63, respectively, when the alkalinity is 232 and 3000 mg/L as CaCO_3_. As the alkalinity increases, a stronger buffering capacity prevents the pH from dropping. Therefore, under higher alkalinity, struvite crystallization and aggregation processes can last longer and lead to a higher PO_4_–P recovery efficiency.

While at a pH of 9.0, it shows a decreasing trend of phosphate removal with the increase of alkalinity in the wastewater in the previous study^39^, because the co-existing carbonate can react with the Mg^2+^ and form more magnesite at a higher pH.

The effect of lignin addition under different alkalinity was also investigated and the result is shown in Fig. 2b. Although the PO_4_–P recovery efficiency is enhanced with the lignin addition at different alkalinity, the increment of PO_4_–P recovery efficiency decreases as alkalinity increases. Since the PO_4_–P recovery efficiency is already very high at higher alkalinity without lignin addition, the effect of lignin becomes insignificant. Therefore, in this study, lignin is more efficient in enhancing the PO_4_–P recovery efficiency at a lower alkalinity.

##### Compositions of precipitate

**Figure 3:**
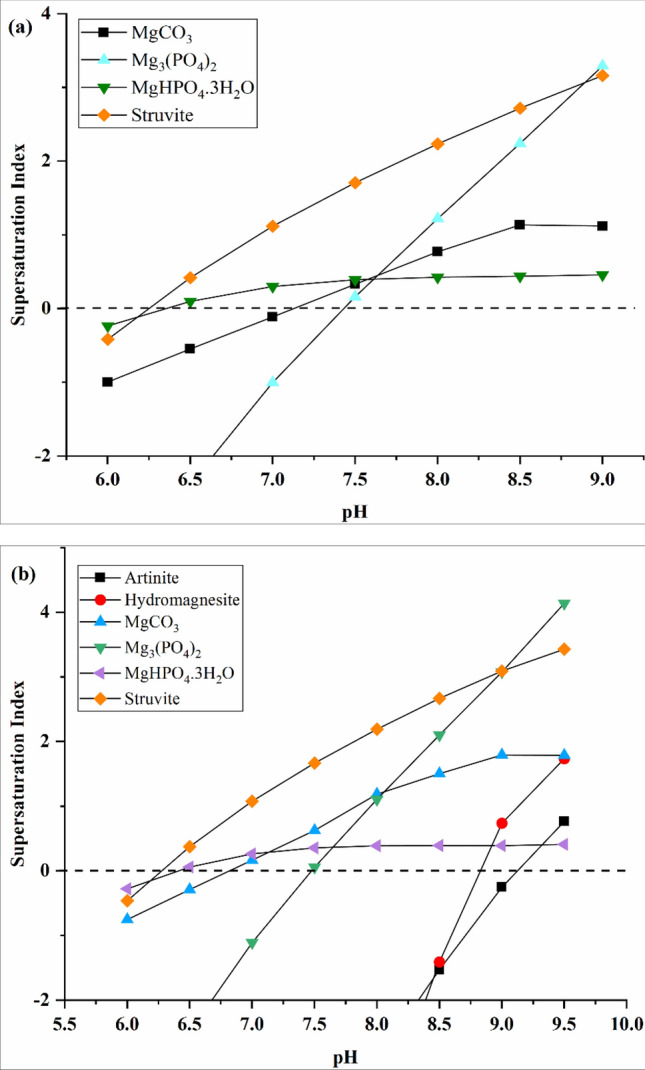
Supersaturation index (*SI*) of different compounds that can be formed from ions present in the synthetic supernatant as a function of pH (without lignin addition) with an alkalinity of (**a**) 1590 and (**b**) 3000 mg/L as CaCO_3_.

The PO_4_–P recovery efficiency increases with the increasing alkalinity, while the composition of the collected precipitation may also change at the higher alkalinity. The prediction of possible precipitates under different alkalinity (1590 and 3000 mg/L as CaCO_3_) by Visual MINTEQ is shown in Fig. 3. As alkalinity increases, the *SI* values of struvite and MgCO_3_ increase simultaneously while the *SI* of Mg_3_(PO_4_)_2_ and MgHPO_4_·3H_2_O decreases, which indicates that struvite is more likely to crystalize under higher alkalinity. Moreover, as pH rises gradually from 8.5 to 9.5 when alkalinity is 3000 mg/L as CaCO_3_ (Fig. 3b), more impurities start to appear such as artinite (Mg_2_(OH)_2_CO_3_·3H_2_O) and hydromagnesite (Mg_5_(CO_3_)_4_(OH)_2_·4H_2_O), reducing the reduction of the produced struvite purity.

**Figure 4:**
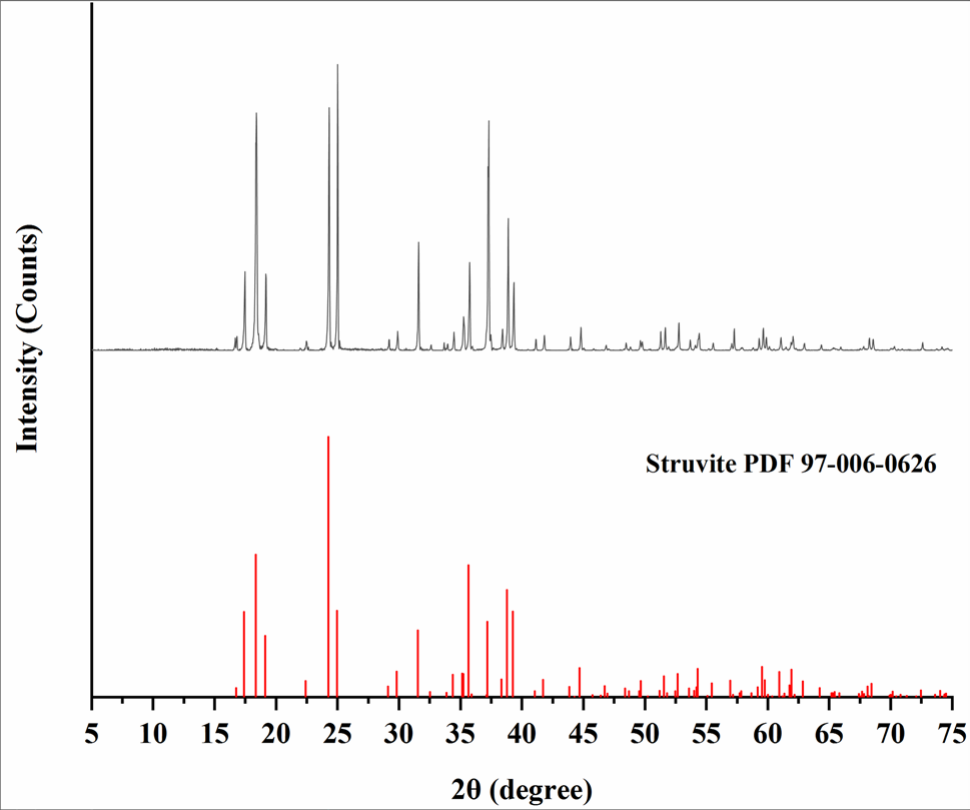
XRD analysis of the solid precipitate at an alkalinity of 3000 mg/L as CaCO_3_ (initial pH = 7.9, Mg/P molar ratio = 1.5, [PO_4_–P] = 250 mg/L, [NH_4_–N] = 1000 mg/L, lignin = 0 g/L).

At pH 7.9, the dominant precipitate may still be struvite for both alkalinity of 1590 and 3000 mg/L as CaCO_3_, as the *SI* of struvite is the highest among all the potential precipitates. It is also proved by XRD analysis of the precipitates at the alkalinity of 3000 mg/L as CaCO_3_, as shown in Fig. 4. Struvite crystal is examined and confirmed by comparison with the struvite reference card from the Inorganic Crystal Structure Database (ICSD). The other precipitates are not detected in this XRD analysis, which is likely due to their small amounts. The other co-precipitates may be determined by the following SEM and EDS analysis.

##### Morphology and elemental analysis

**Figure 5:**
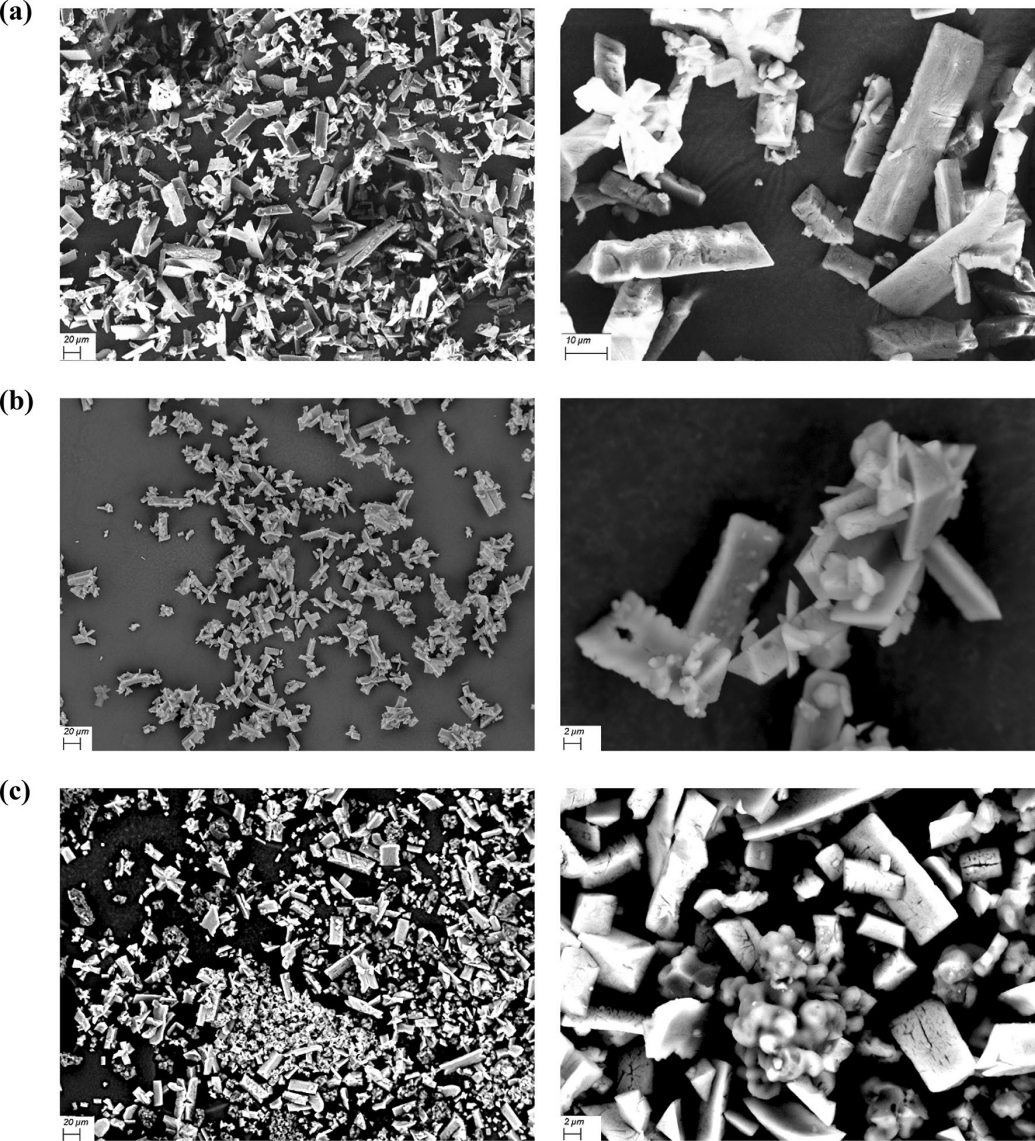
SEM images of the precipitates from the synthetic supernatant at pH = 7.9 with an alkalinity of (**a**) 232 mg/L as CaCO_3_, lignin = 0 g/L, (**b**) 3000 mg/L as CaCO_3_, lignin = 0 g/L, and (**c**) 3000 mg/L as CaCO_3_, lignin = 6 g/L.

The morphology characteristics of the precipitate under various alkalinities were obtained by SEM. Different magnification views for the samples at alkalinity 1590 and 3000 mg/L as CaCO_3_ are shown in Fig. 5.

**Figure 6:**
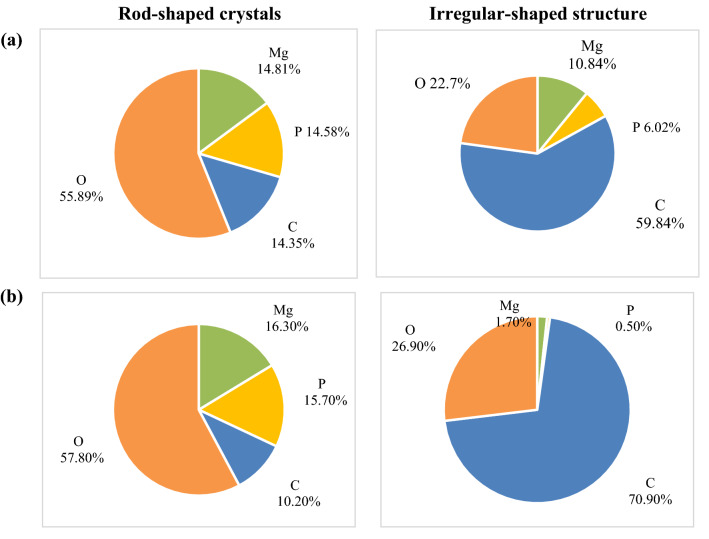
EDS analysis (atom%) of rod-shaped crystal and irregular structure observed in SEM images of the precipitates based on point analysis from the synthetic supernatant (pH = 7.9, alkalinity = 3000 mg/L as CaCO_3_) with lignin of (**a**) 0 g/L and (**b**) 6 g/L.

Without lignin addition, under higher alkalinity, the struvite still remains its typical orthorhombic morphology while some irregular cluster crystals are attached to the struvite surface. The EDS results shown in Fig. 6a indicate that the cluster crystals are likely to be carbonate compounds, such as magnesium carbonate (MgCO_3_) simultaneously co-precipitated with struvite, which is consistent with the precipitation tendency predicted by Visual MINTEQ (Fig. 3).

Compared to struvite at the alkalinity of 232 mg/L as CaCO_3_, the size of the struvite produced under higher alkalinity 3000 mg/L as CaCO_3_ is much smaller (decreasing from 30 to around 10 μm), which is consistent with the previous study^38^. A possible reason may be that higher alkalinity stabilizes the pH at a relatively high level which leads to a higher supersaturation, thus the nucleation rate is higher than the growth rate and more fine particles form^45,46^. Therefore, although higher alkalinity results in higher PO_4_–P recovery, more fine crystals and co-precipitates are likely to form, which reduce the purity of the struvite collected.

Compared to the precipitate without lignin addition at the alkalinity of 3000 mg/L as CaCO_3_ (Fig. 5b), with 6 g/L lignin addition (Fig. 5c) the lignin-struvite clusters are observed and nearly all the crystals are orthorhombic, which indicates that the purity of struvite is higher. The EDS (Fig. 6b) also shows that Mg/P atomic ratio of the crystals are all around 1.0, which confirmed that lignin still reduces the potential of co-precipitates under higher alkalinity.

### Struvite precipitation from the real wastewater

The phosphorus-rich biosolid digestate supernatant, which was collected from a digester sludge thickening lagoon in the City of Edmonton, was used to evaluate the performance of phosphorus recovery efficiency. The lagoon supernatant was pretreated on-site in an Ostara facility for phosphorus removal. The characteristics of this kind of wastewater are shown in Table 1^47,48^.

**Table 1:** Composition of the pre-Ostara biosolid digestate supernatant.

Parameters	Unit	Mean ± SD
TSS	g TSS/m^3^	305 ± 107
NH_4_^+^	mg N/L	891.9 ± 79
NO_2_^−^	mg N/L	0.4 ± 0.7
NO_3_^−^	mg N/L	0.2 ± 0.7
Free ammonia	mg NH_3_–N/L	13.2 ± 1.2
Na	mg Na/L	108.5 ± 1.9
K	mg K/L	310.6 ± 10.8
Si	mg Si/L	24.1 ± 0.07
Ca^43^	mg Ca/L	15.7 ± 0.1
Cr^52^	µg Cr/L	83.4 ± 3.3
Cr^53^	µg Cr/L	65.5 ± 1.7
Cu	µg Cu/L	147.7 ± 10.2
Ni	µg Ni/L	127.6 ± 12.0
Zn^64^	µg Zn/L	100.2 ± 14.1
Zn^66^	µg Zn/L	103.7 ± 11.0
PO_4_^3−^	mg PO_4_–P/L	235 ± 15.4
Alkalinity	mg CaCO_3_/L	3080 ± 51.9
pH	–	7.87 ± 0.15
COD	mg/L	623.0 ± 64.4
BOD_5_	mg/L	218.0 ± 26.0

When the real biosolid digestate supernatant under its origin pH 7.9 is used, the PO_4_–P recovery efficiency with or without lignin addition is 98.67% ± 0.83% and 95.55% ± 1.25% (lignin = 6 g/L or 0 g/L, Mg/P molar ratio = 1.5), respectively, which is much higher than the PO_4_–P recovery efficiency when using the synthetic wastewater. The composition of the real biosolid digestate supernatant is relatively complex compared to the synthetic wastewater. Therefore, the content of the total suspended solids (TSS) might also be a possible factor resulting in the higher PO_4_–P recovery efficiency.

**Figure 7:**
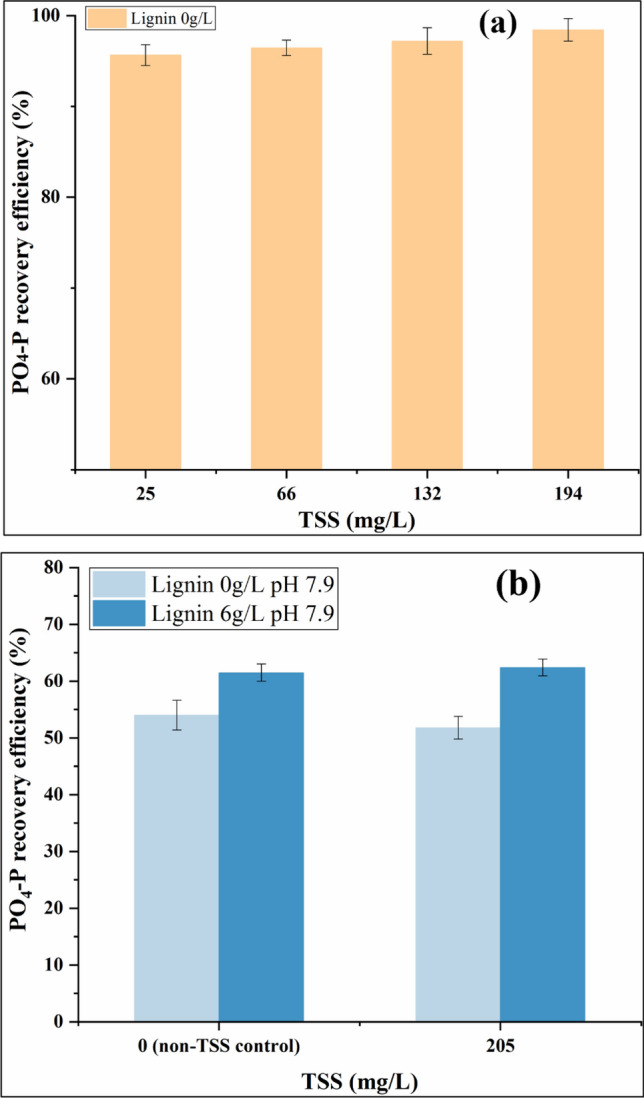
Impact of total suspended solids (TSS) on PO_4_–P recovery efficiency for (**a**) real biosolid digestate supernatant, (**b**) synthetic biosolid digestate supernatant (Mg/P molar ratio = 1.5, [PO_4_–P] = 250 mg/L, [NH_4_–N] = 1000 mg/L).

To figure out if the content of TSS might contribute to a high phosphorus recovery from the real wastewater, different concentrations of TSS were added to the real biosolid digestate supernatant and the phosphorus recovery from these mixtures were tested. Different dewatering methods and operation procedures may cause variable TSS concentrations in municipal wastewater, which could influence struvite crystallization^37^. Figure 7a shows that under the real biosolid digestate supernatant with different TSS, there are nearly no differences of the PO_4_–P recovery efficiency (*p* > 0.05, statistically no difference). Figure 7b also depicts the relationship between the TSS and phosphorus recovery efficiency with or without the lignin addition at pH 7.9 using the synthetic biosolid digestate supernatant. When the TSS was increased from 0 to 205 mg/L, the change of PO_4_–P recovery efficiency was not significant (*p* > 0.05, statistically no difference), which indicates that in this study under the tested pH range, the PO_4_–P recovery efficiency for both lignin- and non-lignin- struvite precipitation is not interfered by the suspended solids. Moreover, TSS is not the related reason for the higher PO_4_–P recovery efficiency of real pre-Ostara biosolid digestate supernatant.

## Conclusions

The lignin-induced struvite crystallization was investigated from the synthetic wastewaters with different initial PO_4_–P and NH_4_–N concentrations, TSS and alkalinity. The phosphorus recovery of lignin-induced struvite precipitation from the synthetic wastewaters increases with increasing concentrations of PO_4_–P, NH_4_–N or alkalinity. At a relatively lower pH of 7.9, the effect of lignin on enhancing struvite precipitation and PO_4_–P recovery from the synthetic wastewaters is still significant under different PO_4_–P and NH_4_–N concentrations. Moreover, lignin is more efficient to promote PO_4_–P recovery under relatively lower alkalinity and still effective to reduce the potential of co-precipitates formation under higher alkalinity. Also, in this study, the result indicates that increasing TSS concentration to 205 mg/L in the synthetic wastewater does not show the significant effect on phosphorus recovery efficiency. Therefore, the effect of lignin in promoting phosphorus recovery from synthetic wastewaters with different characteristics is confirmed in this study, which widens the scope of using lignin as the seed for promoting phosphorus recovery and recycling from various wastewaters. Compared with the synthetic wastewaters, adding lignin only slightly increases PO_4_–P recovery efficiency from the real biosolid digestate supernatant, which can be ascribed to its high alkalinity (> 3000 mg/L as CaCO_3_). It should be noted that natural wastewaters are relatively more complex than synthetic wastewaters, so the findings observed with synthetic wastewaters may not be directly applicable to natural wastewaters, and more studies on lignin-induced struvite crystallization from different natural wastewaters will be conducted.
